# Aqueous‐alcoholic *Ferulla* extract reduces memory impairments in rats exposed to cadmium chloride

**DOI:** 10.1002/brb3.2285

**Published:** 2021-07-21

**Authors:** Homeyra Fadaei, Soheilaalsadat Mirhosseiniardakani, Asghar Farajzadeh, Seyed Sharokh Aghayan, Moslem Jafarisani, Behzad Garmabi

**Affiliations:** ^1^ Department of Medical sciences Babol Branch Islamic Azad University Babol Iran; ^2^ Clinical Biochemistry Islamic Azad University Ardabil Branch Ardabil Iran; ^3^ Clinical Research Development Unit, Imam Hossein Hospital Shahroud University of Medical Sciences Shahroud Iran; ^4^ Cellular and Molecular Biology University of New Haven West Haven Connecticut USA; ^5^ Environmental and Occupational Health Research Center Shahroud University of Medical Sciences Shahroud Iran; ^6^ Neuroscience School of Medicine Shahroud University of Medical Sciences Shahroud Iran

**Keywords:** antioxidant, cadmium poisoning, *Ferulla*, neurotoxicity

## Abstract

**Introduction:**

Cadmium (Cd) is the most dangerous heavy metal that is becoming more widespread in nature as a result of industrial activities. One of the toxic effects of Cd on the body is its neurological effect. The mechanism of these effects has been attributed to the induction of oxidative stress. *Ferulla* plant has antioxidant properties. In the present study, the aim was to reduce the toxic effects of Cd on memory impairment in rats by through the consumption of *Ferulla* extract.

**Materials & Method:**

Rats were randomly divided into five groups of six: (1) control group, (2) 300 μM cadmium exposure group, and three treatment groups with doses of (3) 100, (4) 300, and (5) 600 mg/kg.BW of *F. Ferulla* extract after Cd exposure. To induce neurotoxicity, Cd was daily injected peritoneally at a concentration of 300 μM in 1 ml of normal saline for a week. Next, for 3 weeks, the Cd group received 1 ml of normal peritoneal saline, and the treatment groups received *F. Ferulla* extract at concentrations of 100, 300, and 600 mg/kg.BW in 1 ml of normal saline daily for a week. At the end of the treatment period, a water maze was used to assess memory disorders. Malondialdehyde (MDA), glutathione concentration (GSH), and glutathione peroxidase (GPX) activity in nerve tissue were also measured. Morris water maze was also performed after intervention.

**Results:**

Cd‐induced neurotoxicity was shown in Cd groups. MDA, GSH, and GPX have a significant difference in comparison between the Cd and 300, 600 treated groups. MDA has a significant increase (*p* < 0.05), and GSH and GPX have a significant decrease (*p* < 0.05). The results of the Morris water maze showed that the Cd group spent either 300 or 600 more distances and time to find a place to escape, which was significant (*p* < 0.05)

**Conclusion:**

Cd exposure can induce neurotoxicity and disrupt learning and memory. On the other hand, *Ferulla* extract can improve learning and memory in Cd‐induced neurotoxicity model via induced antioxidant defense system.

## Graphical abstract

Cadmium has a neurotoxic effect and caused memory and learning disruption. Ferulla has an antioxidant effect and can prevent cadmium‐induced neurotoxicity.

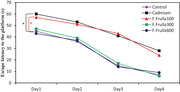

## 1 INTRODUCTION

Cadmium (Cd) and its salts are among the most common environmental pollutants that are increasing in intensity due to the development of industrial processes and activities (Tanu et al., 2018). This heavy metal has very toxic effects on the body of organisms, especially humans (Tanu et al., 2018; Yu et al., 2018). Human beings are daily exposed to this ubiquitous environmental toxicant through nonoccupational sources, including food and water ingestion, and occupational sources, mainly in processes involving heating Cd‐containing materials, such as the production of alloys and batteries (da Silva et al., 2020).

According to the Toxic Substance and Disease Registry (ATDSR), Cd is the seventh most toxic heavy metal (Branca et al., 2018). Exposure to Cd occurs in a variety of ways, including access through the gastrointestinal tract. With the increase of smoking, the respiratory tract has also become one of the methods of exposure (Goering et al., 1994; Klaassen et al., 2009; Waalkes, 2003). Continuous exposure to Cd leads to its accumulation in vital tissues, causing dysfunction (Jacobo‐Estrada et al., 2017; Wajdzik et al., 2017). Harmful effects of Cd exposure have been demonstrated in some studies, especially in mice (Halder, Kar, Galav et al., 2016; Halder, Kar, Mehta et al., 2016). Major adverse effects of Cd exposure have been attributed to oxidative stress (Fongsupa et al., 2015; Skipper et al., 2016). Changes of intracellular calcium (Ca) levels is another pathogenic effect of Cd (Berridge et al., 2000) The toxic effects of Cd are highly dose‐dependent and vary from cell damage, cell death to organ dysfunction (Lopez et al., 2003; Pacini et al., 2009). Toxic effects from exposure to Cd have also been attributed to its long half‐life and ability to accumulate in tissues for long periods of time (Andrade et al., 2017).

Cd damage has been identified in tissues of the liver, kidneys, lungs, the reproductive system, as well as the brain and nervous system in Chong et al. (2017). It was also demonstrated that the itai‐itai disease occurred in Japan due to Cd exposure (Sun et al., 2021).

These complications have shown a wide range of symptoms regardless of the patient's age, such as taste disturbance, neurological and peripheral disorders (testicular disorder, skeleton problem, nephropathy (da Silva et al., 2020)), mental fatigue, learning disabilities, as well as motor and behavioral disorders (Wang & Du, 2013). In addition, Cd‐induced neurotoxicity in several neurodegenerative diseases such as Alzheimer's (AD), Parkinson's (PD) (Chin‐Chan et al., 2015), multiple sclerosis (MS) (Sheykhansari et al., 2018), and encephalomyelitis myalgia (Pacini et al., 2009), has been reported. Cd enters mammalian cells through both active (ABC family carriers and receptor‐dependent endocytosis) and passive (nonspecific carriers and channels) pathways, and its metabolism appears to be similar to Ca metabolism, due to its dual capacity (Choong et al., 2014; Thévenod, 2010).

The most important mechanism in the development of Cd‐induced toxicity that is accepted by researchers is the induction of an oxidative stress system centered on reactive oxygen species (Jomova & Valko, 2011).

Numerous reports of oxidative condition modification by the antioxidant system and thiol groups in the presence of low concentrations of Cd (1–10 μM) are available, and due to the high tendency of Cd to thiol groups, metallothionein and glutathione are the main removers of Cd (Sandbichler & Höckner, 2016). Since Cd can easily cross the blood–brain barrier membrane, neurotoxic effects will be inevitable (Flora et al., 2008).

Previous studies reported antioxidant responses and the role of *Moringa oleifera* leaf extract for the mitigation of Cd‐stressed *Lepidium sativum* L (Khalofah et al., 2020). The *F. Ferulla* plant, as a native plant of Iran, has strong antioxidant properties. The extract of this plant is rich in flavonoid and alkaloid compounds, with antioxidant properties that can increase the body's antioxidant capacity (Lahazi et al., 2015). We also reported protective effects of *F. Ferulla* against Pb‐induced neurotoxicity (Khastar et al., 2019). The aim of present study was to reduce the effects of neurotoxicity in the brains of Cd‐exposed mice using *F. Ferulla* extract.

## 2 MATERIALS AND METHODS

### 2.1 Preparation of the extract

The *F. Ferulla* leaves were purchased from perfumers from Shahroud, Semnan province, Iran, in the spring, and were approved by a botanist, then dried in the shade.

To provide a hydro‐alcoholic extract, the grounded dried leaves (50 g) of the plant were drenched in ethanol (50%) for 48 h using a paper filter. The extract was then filtered. A rotary vacuum evaporator was used to dry the extract (Norouzi et al., 2019).

### 2.2 Animals and treatments

In order to conduct the present experimental study, 30 male Wistar rats with an average weight of 245 ± 20 g were used. The samples were placed in the laboratory at a temperature of 22°C and free access to water and food was given for a week, before the start of the experiment, for the purpose of their adaption to the environment. It is noteworthy that ethical points regarding the care of laboratory animals were observed throughout the work.

Rats were randomly divided into five groups (*n* = 5):

1) Control group, which received 1 ml normal saline i.p.
2) Cd group, which received 300 μM Cd volume of 1 ml normal saline (Khastar et al., 2019).
3–5) Cd plus extract, three treatment groups at doses of 100, 300, and 600 mg/kg BW of *F. Ferulla* extract (Sani et al., 2010). To induce neurotoxicity, Cd was injected peritoneally at a concentration of 300 μM in 1 ml of normal saline for a week. Then, for 3 weeks, the Cd group received interaperitonealy, 1 ml of normal saline, and the treatment groups received *F. Ferulla* extract at concentrations of 100, 300, and 600 kg BW daily dissolved in 1 ml of normal saline. At the end of the treatment period, a water maze was used to assess memory disorders. Malonedialdehyde and glutathione concentration, and glutathione peroxidase activity in the hippocampus were also measured as described below.

### 2.3 Morris water maze

In order to evaluate the learning and spatial memory of the rats, a Morris water maze was used after the treatment of the rats in the abovementioned manner. The water maze was a black cylindrical water tank with a diameter of 142 cm and a height of 80 cm, filled with water of 20 ± 2°C, slightly more than half full. A black metal platform with a diameter of 15 cm was placed in the center of one of the quarters of the circle, at a depth of 1 cm. This platform was only for the animal to escape from the water, and was placed on a pedestal at the bottom of the tank. The room where the maze was located had additional objects and signs installed, such as signs, posters, shelves, and so forth. The room was completely dark. Each rat underwent four trials over 4 days. To learn the location of the platform from a range that was randomly determined by a computer program, the rat would enter the tank facing the wall, giving the rat a minute to find the platform, otherwise it would be led to the platform.

After each experiment in which the rat found the platform, it was allowed to sit on the platform for 30 s and examine the surroundings. Then, the next trial began. At each test interval, the animal was returned to the cage for 30 s. A video camera was mounted on top of the tank to film the animal during the test. The obtained information was transferred to the computer and analyzed using a tracking software. In each experiment, various indicators such as time taken to find the platform in terms of seconds, distance traveled in centimeters, swimming speed in centimeters per second, and stay‐time in each quarter of the circle, were analyzed. Each day after the test, the rats were dried and warmed gently with a towel and then returned to their cages. After a 4‐day training period, the platform was removed on the fifth day, each rat was allowed to search the tank site for 1 min. The percentage of distance and time that it swimmed in the platform area during this 1 min, and the number of times it entered the platform area were measured.

### 2.4 Tissue collection and evaluation of oxidative stress

To evaluate oxidative stress indices, the following were measured in nerve tissue: Malondialdehyde and glutathione concentration, and glutathione peroxidase activity. At the end of the study, the rats were anesthetized using ketamine and zylosin, and then euthanized; their hippocampi were harvested. Immediately, its wet weight was measured and transferred to a liquid nitrogen tank. Next, using commercial kits (Biovision, USA), the amounts of malonedialdehyde (MDA), glutathione (GSH), and glutathione peroxidase (GPX) were determined in homogenized hippocampi as per kit instructions.

### 2.5 Statistical analysis

One‐way analysis of variance was done to compare the differences between the groups. Tukey Post test was then used to examine the pairs of groups. Finally, the data were considered as mean ± standard deviation (Mean ± SD) for each group, and in all cases *p* < 0.05 was calculated as the level of significance.

## 3 RESULTS

**FIGURE 1:**
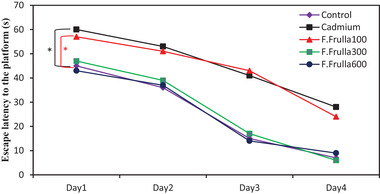
Comparison of delay in finding the platform during the 4‐day training period

There was a significant difference between Cd and control groups in terms of delay in finding the platform during the 4‐day training period. The Cd group was able to find the platform notably later (*p* < 0.01). This delay was also present in the treatment groups, and was significant in the treatment group with the dose of 100 mg/kg, compared to the control (*p* < 0.05), unlike the other treatment groups, as shown in Figure 1.

**FIGURE 2:**
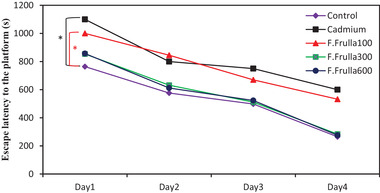
Comparison of traveled distance to find the rescue platform

In this regard, the Cd group rats traveled more distance to find a rescue platform, which was significant compared to the control group (*p* < 0.01). In addition, the treatment group with the dose of 100 mg/kg, compared with the control group, traveled more distance to find a rescue platform (*p* < 0.05), whereas in the other two treatment groups (300, 600) it was not significant, as indicated in Figure 2.

**FIGURE 3:**
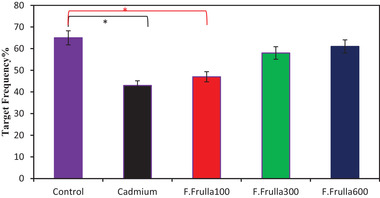
Comparison of the stay‐time of rats within the platform

On day 5, after the removal of the platform and performance of the test, the stay‐time of rats within the platform in the Cd group and the treatment group at a dose of 100 mg/kg was significantly lower in comparison to the control group (*p* < 0.01), whereas in the treatment groups of 300 and 600 mg/kg, there was no significant difference (*p* < 0.05), as shown in Figure 3.

**FIGURE 4:**
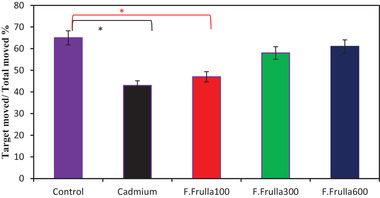
Distance traveled to find the location of the removed platform on the fifth day

In terms of distance traveled to find the location of the removed platform on the fifth day, the Cd group and the 100 mg/kg dosed‐treatment group traveled longer to be present at the site, which was statistically significant compared to the control group (*p* < 0.01). There were no significant differences between the treatment groups with the dose of 300 and 600 mg/kg and the control group (Figure 4).

**FIGURE 5:**
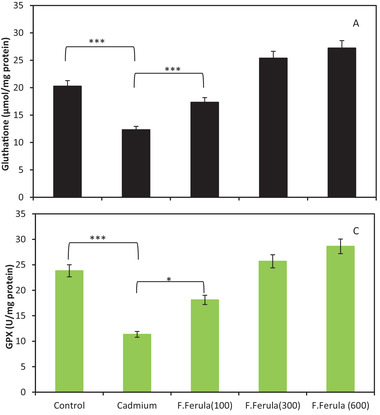
Examination of hippocampus oxidative stress indices

Examination of hippocampus oxidative stress indices showed significant differences among the Cd group, the treatment group at a dose of 100 mg/kg, and the control group. The results showed elevated malonedialdehyde concentration (*p* < 0.01) (Figure 5a), and decreased glutathione concentration (Figure 5a), and glutathione peroxidase (Figure 5c) activity (*p* < 0.05) in Cd group and the treatment group with the dose of 100 mg/kg. Oxidative stress indices in the treatment groups of 300 and 600 mg/kg were not changed significantly, compared to the control group (Figure 5).

## 4 DISCUSSION

What is known today is that Cd can enter the central nervous system (CNS) by increasing blood–brain barrier permeability (Shukla & Chandra, 1987); its accumulation in nerve tissues leads to cell dysfunction and, consequently, occurrence of diseases such as AD, PD, and ASD (autism spectrum disorder) (Barnham & Bush, 2008; Gonçalves et al., 2010). The results of previous studies show that Cd, after penetrating the cell, interacts with voltage‐dependent Ca channels and impairs Ca homeostasis (Snutch et al., 2013). In addition, Cd causes cell damage and apoptosis by inducing the release of cytochrome C from mitochondria (Polson et al., 2011)

Since the CNS is rich in lipids, the formation and increase of any free radicals, and then oxidative stress, can lead to the beginning of lipid oxidation and the production of malondialdehyde (El‐Tarras et al., 2016). In addition to lipid oxidation, macromolecules such as proteins oxidize, resulting in the formation of l‐carbon groups, which are associated with protein dysfunction, and consequently, cell damage, as an indicator of neurotoxicity.

In the present study, it is shown that exposure to Cd significantly increases the time and distance traveled by rats to find the platform on training days, and even the location of the platform on the last day of the Morris water maze test, indicating damage to areas related to memory and learning in the nervous system. Groups treated at high doses were significantly more successful than the Cd group in finding the lifeline in the shortest time and distance. Even after the removal of the platform, they had a good memory of the location of the platform, and the duration of their stay time and the frequency of their presence at the location of the removed platform decreased. This could indicate a possible increased risk of AD in people exposed to Cd (Barnham & Bush, 2008).

Also, an increase in malonedialdehyde levels was observed in the Cd‐exposed group, while the concentration of glutathione and glutathione peroxidase activity as antioxidant defense elements decreased. In addition, increased cell oxidative damage, and memory and learning power impairment were also observed in rats exposed to Cd. The reason for observing these results can be explained in the tendency of Cd to sulfhydryl groups and therefore, the decrease in glutathione concentration and glutathione peroxidase activity.

The *Ferulla* plant is native to Iran. Numerous studies have shown that its extract are compounds containing sulfhydryl group. Also, terpenes are a major part of its active compounds. These groups have antioxidant properties (Lahazi et al., 2015), and since the main mechanism of the damage caused by exposure to Cd is oxidative stress, the use of compounds with antioxidant properties is effective in preventing lesions and improving symptoms. Evidence of this claim is the normal behavior of rats with memory impairment in learning about rescue platform and finding it, which was observed in treatment groups 300 and 600. In the Cd group, a disorder caused by nerve damage was observed.

The results of the present study suggest that Cd‐exposure in rats impairs their memory and learning ability due to the neurotoxic effects of the metal. *Ferulla* plant extract, due to its antioxidant properties, was able to act as an antioxidant and neuroprotective agent, and improve the symptoms of Cd‐toxicity. The results of the oxidative stress study confirmed the mechanism of injury, which was observed with antioxidant therapy, along with the improvement of oxidative stress indices, proper memory function, and learning power.

## 5 CONCLUSION

The aim of the present study was to investigate the effect of aqueous alcoholic extract of *Ferulla* plant on memory disorders in Cd‐exposed rats, and to determine its probabilistic mechanism. We found that daily exposure to Cd could impair memory in exposed rats, but administration of aqueous alcohol extract in dose‐dependent *Ferulla* improved memory. These results suggest that the compounds in the extract may be able to counteract the process of memory impairment in Cd‐exposed rats and reduce its effects and improve the memory disorder.

## CONFLICT OF INTEREST

The authors declare that there is no conflict of interest.

## AUTHOR CONTRIBUTIONS

*Investigation*: Homeyra Fadaei. *Conceptualization, data curation, and investigation*: Asghar Farajzadeh. *Data curation and investigation*: Seyed Sharokh Aghayan. *Validation and visualization*: Soheilaalsadat Mirhosseiniardakani. *Methodology and formal analysis*: Behzad Garmabi. *Project administration, writing original draft, writing review editing, and supervision*: Moslem Jafarisani.

### PEER REVIEW

The peer review history for this article is available at https://publons.com/publon/10.1002/brb3.2285.

## Data Availability

The data that support the findings of this study are available from the corresponding author upon reasonable request.
